# Low Prevalence of Amalgam-Associated Lichenoid Lesions in the Oral Cavity: A Prospective Study

**DOI:** 10.7759/cureus.22696

**Published:** 2022-02-28

**Authors:** Pankaj Gupta, Shivani Mallishery, Nikita Bajaj, K Banga, Ashna Mehra, Rajiv Desai

**Affiliations:** 1 Conservative Dentistry and Endodontics, Nair Hospital Dental College, Mumbai, IND; 2 Dentistry, University of Colorado, Colorado, USA; 3 Neuroscience, Garvan Institute of Medical Research, Sydney, AUS; 4 Dental Surgery, Nair Hospital Dental College, Mumbai, IND; 5 Oral and Maxillofacial Pathology, Nair Hospital Dental College, Mumbai, IND

**Keywords:** delayed hypersensitivity reaction, oral cavity, lichenoid lesions, contact allergy, amalgam

## Abstract

Introduction: Amalgam has been the restoration of choice for years, but its popularity has declined due to concerns about aesthetics, mercury toxicity and lichenoid lesions associated with it. Lichenoid reaction is considered to be a delayed hypersensitivity type of reaction and it has been associated with dental materials in general and amalgam in particular.

Materials and Methodology: Two thousand patients having at least one amalgam restoration were examined for signs of lichenoid lesions when visiting the OPD of Conservative Dentistry and Endodontics at the Nair Hospital Dental College in Mumbai, India. Indirect spatial correlation to the amalgam restoration and the same were recorded. Descriptive analysis was used.

Results: Three (0.15%) out of 2000 patients with amalgam-associated lichenoid lesions showed complete resolution of lesions after the replacement of the restorations.

Conclusion: Amalgam associated lichenoid lesions have a low prevalence and should not be a contraindication to its use in routine restorative dental practice. Patch tests and biopsies have questionable diagnostic and prognostic value. Identification of the lesions should be made after the elimination of all other causative factors for the presenting symptoms. A close spatial association of the lesion to amalgam and the regression of symptoms after its removal should be considered as confirming the diagnosis.

## Introduction

The use of amalgam as a restorative dental material has the longest history dating back to 659 AD [1] and has enjoyed popularity as a restorative material of choice [2] for decades which can be attributed to its durability as indicated by numerous studies [3,4]. In recent times, the use of amalgam has decreased considerably owing to concerns about the environment [5,6] and mercury toxicity along with increasing demand for aesthetic restorations by the patients [7,8]. Another health concern attributed to amalgam, in particular, and dental materials, in general, is a delayed hypersensitivity type of reaction similar in clinical and histological appearance to lichen planus, termed oral lichenoid lesion (OLL) [9-11]. Numerous studies and a systematic review has been published on the subject of OLL and its association with amalgam restorations [12]. All the studies published to date were retrospective and had a small sample size [7,8,10]. The current study was intended to be an observational prospective study to determine the prevalence of OLL in patients with amalgam restorations.

## Materials and methods

Patients visiting the OPD of Conservative Dentistry and Endodontics at the Nair Hospital Dental College in Mumbai, India, were examined from February 2017 to March 2018. Patients with at least one amalgam restoration (age of the restoration > six months from date of observation) were included in the study. The patient was informed about the purpose of the study and given a patient information sheet. Patients with any alteration of the buccal and/or tongue mucosa were examined with a detailed case history and clinical examination. Patients with a history of various systemic diseases like diabetes, hypertension, or thyroid disorders were excluded from the study. Informed consent was taken from all the patients. The study was approved by the Institutional Ethics Committee of Nair Hospital Dental College (IRB Approval No.EC-44/CONS-04ND04/2016).

The diagnostic criteria for positively identifying the lesion as an amalgam-associated lichenoid lesion were adopted from that recommendation of the world workshop of oral medicine IV [13] which included the direct topographical relation of the lesion to the offending material otherwise clinically and histologically indistinguishable from oral lichen planus. Another diagnostic criterion suggested was the resolution of the lesion after removal of the presumed causative restorative material, which was followed in the present study. The additional diagnostic and identification criteria adopted from the study by Cobos-Fuentes MJ et al. specifies that the lesion is asymmetric [14].

The clinicians who carried out the actual examination of the patients were supervised and calibrated by a specialist in the field of oral pathology. The oral cavity was examined for signs of alterations in the mucosa under the dental chair headlight with a sterile mouth mirror after drying the area with gauze. Any abnormality in the mucosa was recorded and further examined by an appropriate specialist along with a detailed case history of the same.

## Results

Two thousand patients were examined during this study between February 2017 to March 2018. Patients who fit the inclusion criteria were examined for any alterations in the adjacent oral mucosa. The recorded results were analysed using descriptive analysis.

Of the patients examined, 55.6% (n=1112) were male and 44.4% (n=888) female. The mean age of the examined population was 43.45 years. The average number of amalgam fillings, surfaces covered by amalgam and the age of amalgam restorations were 2.82, six and nine, respectively. 

Three (0.15%) out of 2000 patients were clinically diagnosed as suffering from a lichenoid lesion caused by amalgam restoration based on the diagnostic criteria mentioned above. All these patients presented a white reticular appearance on an erythematous background. These patients gave a positive history of burning sensation and discomfort exacerbated by spicy food in the areas adjacent to the amalgam restorations soon after its placement. The most commonly affected areas were the buccal mucosa followed by the tongue. These lesions were confined to the area in direct contact with the amalgam restorations. All patients demonstrated complete resolution of the lesions after replacement with an alternative restorative material.

**Figure 1:**
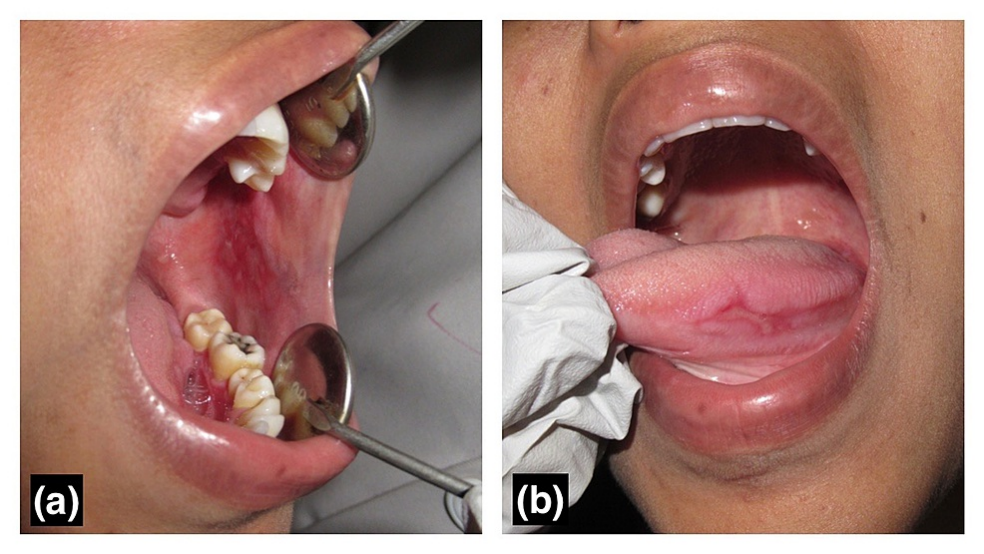
Clinical image shows patient with amalgam-associated lichenoid lesion before removal of amalgam restoration (a) Lesion on the buccal mucosa, (b) Lesion on the tongue

**Figure 2:**
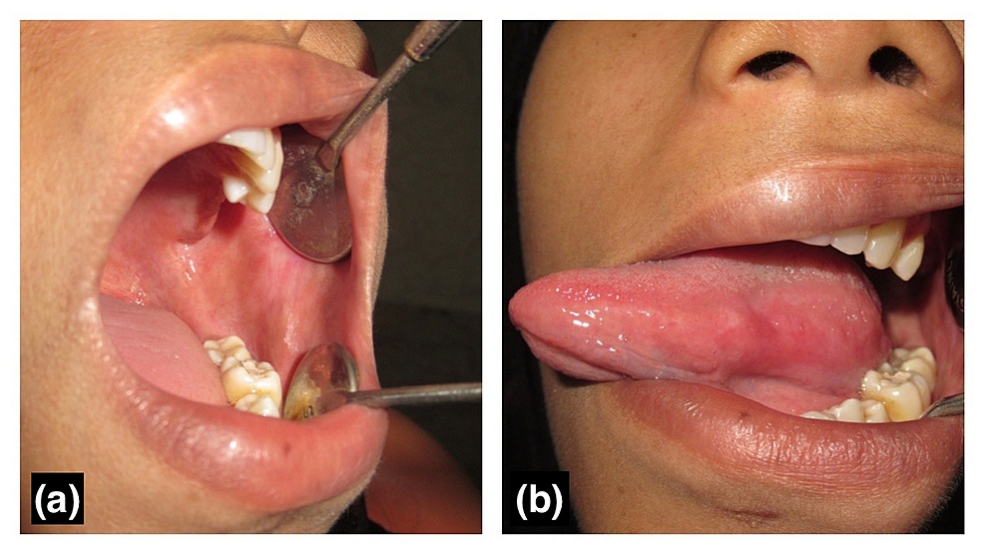
Clinical image shows one-month follow-up after removal of amalgam restoration. (a) No lesion on the buccal mucosa, (b) No lesion on the tongue

The pre and post-amalgam removal photographs of a patient with amalgam-associated lichenoid lesions can be seen in Figure 1 (before removal) and Figure 2 (one month after amalgam removal). Lesions other than lichenoid lesions observed in the course of the study are tabulated in Table 1.

**Table 1:** Lesions other than lichenoid lesions observed during the study

Sr. no.	Lesions	Percentage (out of 2000 patients)
1.	Lichen planus	1.45% (29)
2.	Linea alba	0.4% (8)
3.	Hyperkeratosis due to occlusal disturbances caused by improper counters of the restorations	0.7% (14)
4.	Cheek bite	1% (20)
5.	Aphthous ulcers	0.2% (4)
6.	Leukoplakia	0.25% (5)

## Discussion

Dental amalgam has been successfully used as a restorative material because of its durability and technique insensitivity [15,16]. It has the longest clinical track record and serves as a golden standard for other restorative materials to compare with [17]. Over the last few decades, the popularity of amalgam restorations has declined primarily because of environmental concerns [5,6] along with aesthetic concerns of the patients [18,19] and concerns about the use of mercury [20-22].

Another reason for the decline in the use of amalgam is the allergic reaction of the oral mucosa to one or more of its components, most commonly mercury. This particular hypersensitivity reaction has been documented in numerous studies, though most of the studies had common limitations [8,14,16]. To the best of the authors' knowledge, no study to date has been prospective. Therefore, the actual prevalence of lichenoid lesions caused by amalgam is not known. Previous studies investigating amalgam-associated lichenoid reactions had a small sample size of not more than 150 patients and included patients with amalgam restorations and lichenoid type lesions present at the time of observation. These studies then proceeded with patch test or biopsy or both and replacement of the amalgam restorations [7,23-25] after which the results were documented.

A systematic review in 2004 reported 19 studies where patch test was done and amalgam replaced by alternative material and healing was observed [10]. The total number of patients in the studies reported ranges from four to 131. Except for one study, none of the studies reported complete resolution of the lesions in all the subjects with a positive patch test. The study which reported a complete resolution had a sample size of seven patients only. Therefore, it can be concluded that a positive patch test is not a fool-proof method to predict the prognosis of lichenoid lesions. Similar observations about the utility of patch tests have been made in other studies also [1,13,26]. The world workshop of oral medicine IV also recommends that a patch test has utility in assessing an alternative filling material rather than determining the component of amalgam restoration to which the patient is allergic [13]. No patch test was performed in the present study because of the inconclusive evidence of its usefulness in predicting the prognosis of the lesions, though it can be considered for patients with multiple amalgam restorations who are suspected of having lichenoid lesions for economical reasons.

Many lesions that had a presentation similar to the oral lichenoid lesion were observed during the study. These included lichen planus, linea alba, hyperkeratosis due to improperly contoured restorations, cheek bite, aphthous ulcers, and leukoplakia. If any doubt remains in the mind of the attending clinician, an opinion from an appropriate expert in the field should be sought before arriving at the diagnosis of lichenoid lesions.

Furthermore, lesions on the tongue and buccal mucosa may have been caused by improper contours of the restorations which resolved after the restoration was removed and replaced with a well-contoured restoration. Therefore, the mere resolution of the lesion after replacement of the amalgam restoration with an alternative material cannot establish the cause and relationship to lichenoid lesions. The clinician should not be too eager to label a lesion as amalgam-associated lichenoid lesions before considering the above-mentioned factors.

In the present study, all patients who demonstrated healing had symptoms that started soon after the placement of amalgam restorations. Therefore, the history of the initial appearance of symptoms can give a definite clue to the etiology of the symptoms. To date, no studies have reported differentiating histopathological characteristics between oral lichen planus (OLP) and oral lichenoid lesions (OLL) [27,28]. And so, the use of a biopsy for distinguishing between OLP and OLL is of no diagnostic and prognostic use and should be reserved for cases that show abnormal presentations. Similar conclusions have been drawn in other studies as well [14,29]. Hence, a biopsy was not included for the diagnosis of OLL.

Confusion persists between OLP and OLL as both demonstrate similar clinical and histological characteristics. Histological examination alone is found to be inconclusive in establishing an accurate diagnosis in the cases. The basic distinguishing factors between OLL and OLP are: (1) OLP lesions are mostly bilateral with a more or less symmetrical distribution on both sides which is in contrast to lichenoid lesions that are unilateral; (2) Lichenoid lesions have a direct topographical correlation to the causative restorative material and are restricted to the area in direct contact to the restoration in contrast to lichen planus which has no such correlation to a restorative material; (3) If after elimination of other causative factors, a lesion in contact with the restorative material heals after its replacement, it is in all probability a lichenoid lesion.

## Conclusions

Though allergic reactions to amalgam or one of its components is a well-established fact, the present study found the prevalence low. The existence of an allergic reaction to amalgam or its components should not deter the clinician from using amalgam in cases where it is indicated as it provides a durable, strong and relatively inexpensive restorative option.

Patients demonstrating clear signs of OLL only, along with thorough case history, should be evaluated for replacement of amalgam to check for healing. No further treatment is required for the same. In the case of non-healing even after the removal of amalgam, other more serious causes for the presentation should be investigated. The present study advocates that amalgam-associated lichenoid lesions should not be considered as a contraindication for its usage as the prevalence of these reactions is low.
